# Osimertinib for compound *EGFR* exon 19 deletion/T790M mutated lung squamous cell carcinoma

**DOI:** 10.1111/1759-7714.13431

**Published:** 2020-07-15

**Authors:** MuYun Peng, QiuYuan Wen, Xia Wu, FengLei Yu, WenLiang Liu

**Affiliations:** ^1^ Department of Thoracic Surgery The Second Xiangya Hospital of Central South University Changsha China; ^2^ Hunan Key Laboratory of Early Diagnosis and Precise Treatment of Lung Cancer The Second Xiangya Hospital of Central South University Changsha China; ^3^ Early‐Stage Lung Cancer Center The Second Xiangya Hospital of Central South University Changsha China; ^4^ Department of Pathology The Second Xiangya Hospital of Central South University Changsha China

**Keywords:** Epidermal growth factor receptor, lung squamous cell carcinoma, osimertinib, tyrosine kinase inhibitor

## Abstract

The role of the epidermal growth factor receptor (*EGFR*) mutation status testing in lung squamous cell carcinoma (SqCC) remains controversial. Evidence of the effectiveness of osimertinib in SqCC with EGFR T790M mutation is limited. Here, we describe a hitherto unreported case of a stage III SqCC patient with compound mutation of *EGFR* exon 19 deletion (19Del) and T790M mutation. Pathological complete tumor response was achieved after treatment with osimertinib. We suggest that *EGFR* mutation testing should be performed in Asian patients who have not been definitively diagnosed with SqCC due to small lung biopsy samples. Osimertinib has shown good efficacy in SqCC harboring a “primary” resistance mechanism (EGFR T790M).

**Key points:**

An unreported case of stage III squamous cell carcinoma with synchronous occurrence of *EGFR* exon 19 deletion (19Del) and T790M mutation. Complete tumor response was achieved after treatment with osimertinib.

*EGFR* mutation testing should be performed in Asian patients who are not definitively diagnosed with SqCC due to small lung biopsy samples. Osimertinib has shown good efficacy in SqCC harboring a “primary” resistance mechanism (EGFR T790M).

## Introduction

Lung cancer remains the leading cause of cancer‐related death worldwide.^1^ Non‐small cell lung cancers (NSCLC) account for about 85% of lung cancers, and of these, approximately 30% are lung squamous cell carcinomas (SqCC).^2^ Epidermal growth factor receptor (EGFR) is a cell‐surface tyrosine kinase receptor that can activate pathways associated with cell growth and proliferation when activated. *EGFR* mutations have become an important therapeutic target for the treatment of nonsquamous NSCLC. EGFR exon 19 deletion (19Del) and exon 21 Leu858Arg point mutation (L858R), which are associated with favorable outcomes in patients treated with EGFR‐tyrosine kinase inhibitors (TKIs), account for 90% of all *EGFR* mutations. EGFR exon 20 Thr790Met point mutation (T790M) was present in approximately 50% to 60% of acquired resistance to EGFR‐TKI.^3^ EGFR T790M mutation can also be detected in a small proportion of tumors before treatment with EGFR‐TKIs.^4^ Third generation TKIs, such as osimertinib, have demonstrated efficacy in patients who develop resistance to first or second generation EGFR‐TKIs due to T790M mutation.^5^

*EGFR* mutation rate is 40% to 50% in lung adenocarcinoma (ADC) cases in east Asian populations.^6^ However, in Asian SqCC patients, incidence of *EGFR* mutation is relatively low, varying from 2% to 13%.^7^, ^8^, ^9^, ^10^ The role of *EGFR* mutation status testing and EGFR‐TKIs in SqCC remains controversial. Some oncology groups recommend *EGFR* mutation testing in all SqCC patients when clinical features indicate a higher probability of an oncogenic driver (ASCO, ACP/IASLC/AMP),^11^ while others recommend it only for patients with SqCC who have never smoked or who have mixed subtypes (ESMO and NCCN).^12^

Here, we report a case of locally advanced SqCC harboring *EGFR* exon 19Del/T790M mutation with a pathological complete tumor response after osimertinib treatment. We also discuss the literature regarding the efficacy of EGFR TKIs in SqCC, as well as their use in the neoadjuvant setting.

## Case report

**Figure 1:**
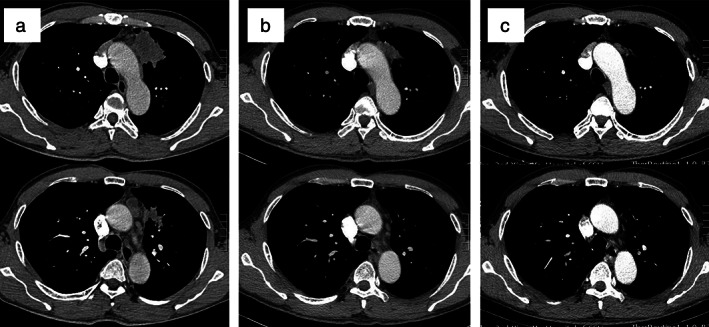
Evaluation by computed tomography (CT) scan. (**a**) At diagnosis. The upper left lobe mass was considered as the primary tumor while the mediastinal mass was considered as station 5 and 6 lymph‐node metastasis. (**b**) One month after osimertinib, both the primary tumor and lymph‐node had decreased in size. (**c**) Three months after osimertinib. The primary tumor had reduced in size remarkably, and the mediastinal mass had disappeared.

**Figure 2:**
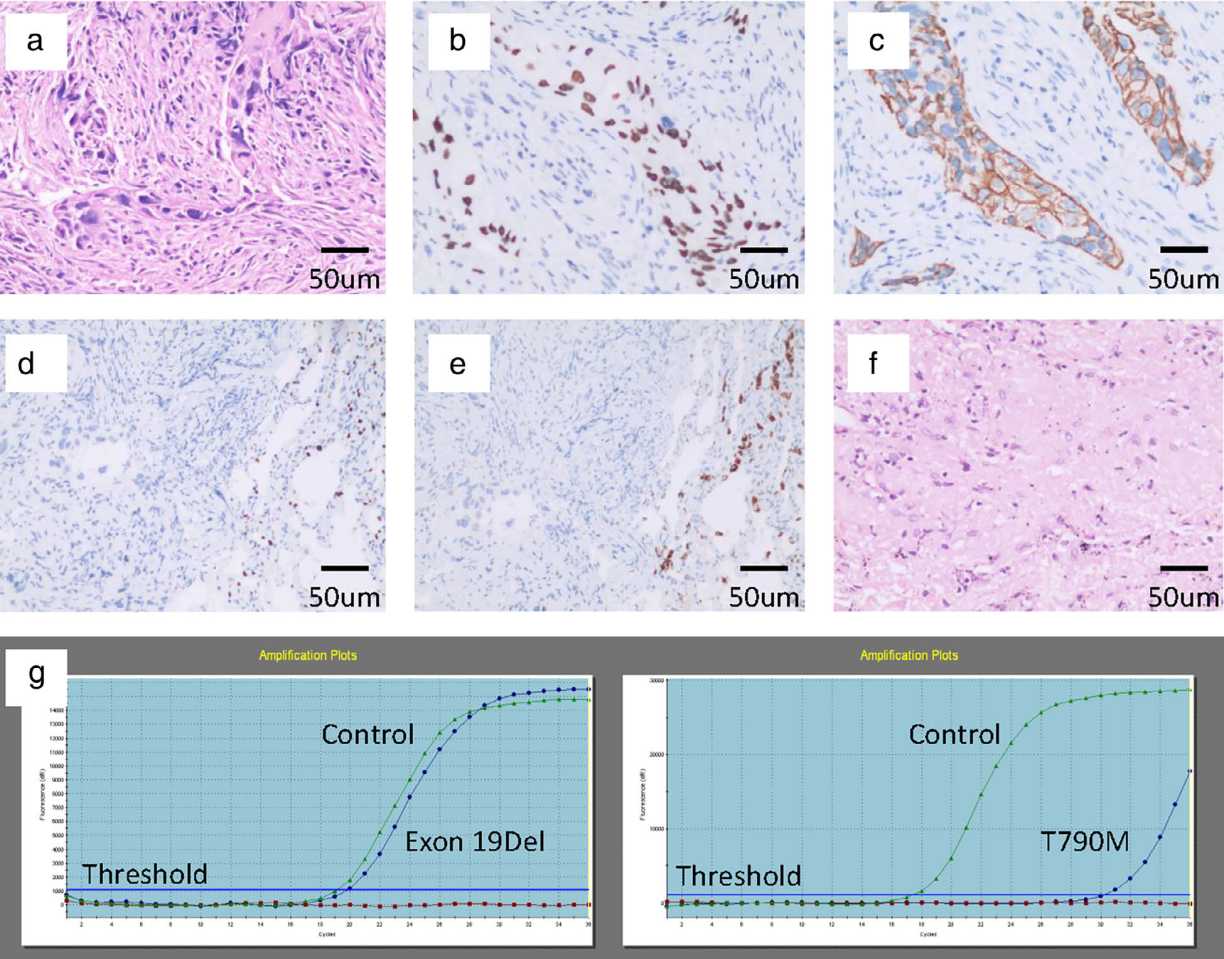
Pathology and amplification refractory mutation system‐polymerase chain reaction (ARMS‐PCR). (**a**) At the time of diagnosis. Hematoxylin and eosin (HE) staining showed neoplastic cells with morphological characteristics of non‐small cell lung cancer (NSCLC). (**b**–**e**) At the time of diagnosis. (**b**) Immunohistochemistry revealed diffuse expression of P40; (**c**) CK (5/6); (**d**) negative expression of TTF‐1; and (**e**) Napsin A, which led to the diagnosis of lung squamous cell carcinoma. (**f**) At the time of surgery. HE staining showed a necrotic area of former cancer tissue, with no residual viable cancer cells. (**g**) At the time of diagnosis. ARMS‐PCR showed coexistence of epidermal growth factor receptor (EGFR) exon 19Del and T790M (magnification: 200x; scale bar: 50 μm).

A 50‐year‐old Chinese Han male, who was a former light smoker with a smoking index of 200, presented with irritable cough and left chest pain in February 2019. Chest computed tomography (CT) scan revealed a pulmonary left upper lobe mass of 5.3 cm with enlarged mediastinal lymph nodes (station 4L, 5 and 6), suggesting lung cancer and the possibility of lymph node metastasis **(**Fig 1a**)**. Fine needle biopsy of the primary tumor confirmed that it was SqCC **(**Fig 2a–e**)**. Molecular status was tested with a panel including common mutated genes in lung cancer. Amplification refractory mutation system‐polymerase chain reaction (ARMS‐PCR) revealed compound occurrence of EGFR exon 19Del and T790M mutation **(**Fig 2g**)**. ALK, ROS‐1 rearrangement and c‐MET amplification were lacking. The patient had no history of malignant tumors and family history. He had normal lung function, no comorbidities, and his clinical stage was cT3N2M0 IIIB (AJCC eighth version). According to NCCN guidelines, the third‐generation *EGFR* inhibitor osimertinib is the preferred first‐line therapy option for patients with metastatic NSCLC with sensitizing *EGFR* mutations. The patient started osimertinib (80 mg per os q.d.) immediately. He had no remarkable adverse effects (AEs). CT scans after one and three months showed a remarkable decrease of the lung lesion and lymph nodes **(**Fig 1b,c**)**. Response evaluation criteria in solid tumors (RECIST) partial response was obtained.

After multiple disciplinary team (MDT) discussion, a VATS left upper lobectomy following systemic lymph nodes resection was conducted. During surgery, regional lymph nodes including lower paratracheal, subaotic, para‐aortic, subcarinal, hilar and interlobar nodes were dissected. The involved mediastinal pleura and left phrenic nerve were resected en bloc, and the left phrenic diaphragm was suspended to the left chest wall to avoid paradoxical movement of the diaphragm. The patient recovered uneventfully and was discharged three days after surgery. Postoperative pathological examination showed coagulative necrosis of the lesion and proliferation of fibrous tissue. No cancer cells were found in the primary lung cancer lesion and lymph nodes **(**Fig 2f**)**. The patient is in follow‐up with adjuvant osimertinib, and recent CT examination eight months after surgery found no evidence of recurrence or metastasis.

## Discussion

Here we describe a hitherto unreported case of a stage III SqCC in a patient with synchronous occurrence of *EGFR* exon 19Del and T790M mutation treated with osimertinib. To date, only a few cases of squamous cell transformation from LADC treated with EGFR‐TKIs with concomitant T790M have been reported.^13^, ^14^, ^15^ There are no reports on the use of osimertinib in SqCC with primary EGFR exon 19Del and T790M compound mutation.

In Asian SqCC patients, incidence of *EGFR* mutation varies from 2% to 13%.^8^, ^9^ According to the updated CAP/IASLC/AMP Molecular Testing guideline, EGFR testing is recommended for adenocarcinomas and mixed lung cancers with an adenocarcinoma component in the setting of lung cancer resection specimens. In the setting of fully excised lung cancer specimens, EGFR testing is not recommended in lung cancers that lack any adenocarcinoma component, such as pure SqCC and pure small cell carcinomas.^16^ However, in the setting of small lung biopsies, adenosquamous carcinoma could be misdiagnosed as SqCC or NSCLC favor SqCC due to undersampling. Ho and colleagues studied 148 small lung biopsy cases with pathological diagnosis of SqCC or NSCLC favor SqCC, and found an *EGFR* mutation rate of 5.2% (7/135) in SqCC and 46.2% (6/13) in NSCLC favor SqCC. They concluded that *EGFR* mutation testing should be performed in Asian patients with SqCC diagnosed from small lung biopsies, especially in never‐smokers and patients with diagnosis of NSCC favor SqCC, which have a high probability of being adenosquamous carcinoma.^17^ Tests of multiple biopsies are helpful for accurate pathological and molecular diagnosis. In our case, the patient was a young, light smoker, and diagnosis came from small biopsy.

Response rates (RR) and median progression‐free survival (PFS) associated with EGFR TKI therapies among SqCC appear to be lower than among patients with adenocarcinoma, with 25% to 43.2% versus 54.4% to 80% for RR and 1.4–5.1 months versus 9–13 months for PFS, respectively.^8^, ^18^, ^19^, ^20^, ^21^ Some reports have argued that *EGFR*‐mutated SqCC have mixed ADC histology due to the diagnostic limitations of small biopsies and intratumoral heterogeneity. Meanwhile, the majority of the population are SqCC with wild‐type EGFR, resulting in inferior RR and PFS. Thus, the sensitivity of EGFR‐TKIs in patients with non‐ADC harboring *EGFR* mutations may depend on the proportion of *EGFR*‐mutated ADC components in the whole tumor.^8^ However, this does not explain why some *EGFR*‐mutated non‐ADC patients respond completely to EGFR‐TIKs, as in our case. The distinction between adenocarcinoma and squamous cell carcinoma can be extremely challenging in some cases. Thus, patients should not be deprived of potentially beneficial nontoxic therapies such as TKIs just on the basis of histology.^19^

Osimertinib is a third generation, irreversible EGFR‐TKI that selectively inhibits both EGFR‐TKI‐sensitizing and *EGFR* T790M resistance mutations. According to the FLAURA trial, osimertinib showed efficacy superior to that of standard EGFR‐TKIs in the first‐line treatment of *EGFR* mutation‐positive advanced NSCLC, with a similar safety profile and lower rates of serious adverse events.^22^ However, evidence of the effectiveness of osimertinib in SqCC with EGFR T790M mutation is limited. Zhang *et al*. reported a case of SqCC with secondary T790M mutation receiving osimertinib, and PFS was less than 10 months. In our case, a pathological complete response was achieved after three months of osimertinib. More cases are needed to clarify the efficacy of osimertinib in SqCC with *EGFR* T790M mutation.

The continuation maintenance therapy with osimertinib is still controversial. Several studies on adjuvant TKI treatment have concluded its safety and feasibility.^23^ However, there are relatively few clinical trials evaluating the efficacy of adjuvant EGFR‐TKIs in SqCC patients. More clinical trials are needed to provide convincing evidence for customized therapy for SqCC patients with *EGFR* mutations.

As far as we are aware, this is the first reported EGFR Exon 19Del/T790M mutation SqCC case with pathologic complete response to osimertinib, which serves as direct evidence of the effectiveness of osimertinib in SqCC. Earlier detection and surgical intervention have been shown to be beneficial for patient outcomes. Further clinical data in patients with SqCC harboring a “primary” resistance mechanism (T790M) to TKIs may be helpful in order to optimize the best treatment for these patients.

## Disclosure

The authors have no conflicts of interest to declare.
